# Practical hints and tips for solution of pseudo-merohedric twins: three case studies

**DOI:** 10.1107/S205698902100342X

**Published:** 2021-04-09

**Authors:** S. R. Parkin

**Affiliations:** a Department of Chemistry, University of Kentucky, Lexington, KY 40506, USA

**Keywords:** pseudo-merohedry, pseudo-ortho­rhom­bic, pseudo-tetra­gonal, pseudo-hexa­gonal, reticular, twinning, HKLF 5 format

## Abstract

Twinning by pseudo-merohedry is a common phenomenon in small-mol­ecule crystallography. In cases where twin-component volume fractions are markedly different, structure solution is often no more difficult than for non-twinned structures of similar complexity. When twin-component volume fractions are similar, however, structure solution can be much more of a problem. This paper presents hints and tips for such cases by means of three worked examples. The first example presents the most common (and simplest) case of a two-component pseudo-ortho­rhom­bic twin. The second example describes structure solution of a reticular threefold pseudo-hexa­gonal twin that benefits from use of an unconventional space-group setting. The third example covers structure solution of a reticular fourfold pseudo-tetra­gonal twin. All three structures are ultimately shown to be monoclinic crystals that twin as a consequence of unit-cell metrics that mimic those of higher symmetry crystal systems.

## Abstract 2

Structure solution for pseudo-merohedric twins having roughly equal individual volume fractions are described in detail via worked examples of varying complexity.

## Introduction   

Twinning in crystallography is the phenomenon by which a crystalline entity may be composed of two or more crystals that are mutually related by precise mathematical relationships. The theoretical aspects of twinning (see *e.g*. Hahn & Klapper, 2006 ▸ and references therein), nomenclature and classification (Donnay & Donnay, 1959 ▸; Nespolo & Ferraris, 2003 ▸; Nespolo, 2015 ▸, 2019 ▸), identification and structure refinement (Herbst-Irmer & Sheldrick, 1998 ▸, 2002 ▸; Parsons, 2003 ▸; Petríček *et al.*, 2016 ▸; Sevvana *et al.*, 2019 ▸) for mol­ecular crystals have been extensively covered in the literature, and need not be repeated here. Nevertheless, a brief introduction is warranted. Within a twin, the component parts are mapped onto each other *via twin operations* (inversion, rotation, reflection) that occur with respect to a *twin element* (point, axis, plane). The family of symmetrically equivalent twin operations, i.e. those that result in the same mapping of component parts onto each other, constitute the *twin law*. This definition distinguishes a true *twin* from a mere *aggregate* (*i.e.* a random conglomerate of two or more pieces). The term ‘twin’ refers to the whole crystalline entity, which is composed of *individuals* or *components* (Nespolo, 2015 ▸) related by the twin law. Although sometimes used inter­changeably, the terms twin *domain* and *individual* are not synonymous. An individual might comprise a single domain or many domains. Domains having identical orientations comprise a single *domain state*. For a macroscopic twin, ‘domain state’ and ‘individual’ are inter­changeable (Nespolo, 2019 ▸). Twins have been classified in different ways (*e.g.* Donnay & Donnay, 1974 ▸; Nespolo & Ferraris, 2000 ▸; Hahn & Klapper, 2006 ▸; Petríček *et al.*, 2016 ▸). Commonly used terms include *twin-lattice-symmetry* (*TLS*), *twin-lattice-quasi-symmetry* (*TLQS*), *twinning by merohedry*, *pseudo-merohedry*, *non-merohedry etc*., with further sub-divisions possible. Definitions of each are given in the Online Dictionary of Crystallography (IUCr ODC, 2021 ▸).

In the diffraction pattern of a twin, the reciprocal lattices of domains comprising an individual superimpose exactly, resulting in the diffraction pattern of that individual. Diffraction patterns from each individual overlap, to an extent determined by the twin law and weighted by their relative irradiated volume fractions, thereby producing the diffraction pattern of the (whole) twin. The degree of overlap from each individual thus readily provides, to a first approximation, a quick and convenient means of assessment. Exact superposition of individual reciprocal lattices occurs for twinning by merohedry. Aside from the special case of twinning by inversion, twinning by merohedry is far more common in minerals and crystals of inorganic compounds than in organic or organometallic crystals. Close, but not symmetrically exact overlap, occurs for twinning by pseudo-merohedry, and is common in mol­ecular crystals. Indexing of any crystal requires finding the link between the reciprocal lattice and the coordinate system of the diffractometer, which takes the form of a mathematical transformation, the *orientation matrix* (Busing & Levy, 1967 ▸). Similar to non-twinned crystals, a (pseudo-)merohedric twin requires just one orientation matrix. In such cases, all diffraction maxima receive a contribution from each individual present. The term *reticular* is used as a modifier for twinning by (pseudo-)merohedry in particular cases where only a well-defined fraction of individual reciprocal lattice points, and hence twin-related diffraction maxima, overlap. The diffraction pattern of a reticular (pseudo-)merohedric twin may also be indexed by just a single orientation matrix, but any individual only contributes to the aforementioned fraction, the reciprocal of the *twin index* (Donnay & Donnay, 1959 ▸), of the observed diffraction maxima. Any deviation from exact overlap for twinning by pseudo-merohedry is qu­anti­fied by *obliquity* (Friedel, 1926 ▸; Donnay & Donnay, 1959 ▸; Wolten, 1966 ▸) or by *twin misfit* (Nespolo & Ferraris, 2007 ▸). Twinning by non-merohedry results in a combination of full, partial, and non-overlapping diffraction maxima. Indexing of such a twin requires a separate orientation matrix for each individual.

For novice or otherwise inexperienced crystallographers, solution and refinement of twinned crystal structures can appear to be a daunting task. Nevertheless, in the absence of other problems such as extensive disorder (*e.g*. Parkin & Hope, 1998 ▸; Hou *et al.*, 2019 ▸) or worse, *e.g.* incommensurate modulation (van Smaalen *et al.*, 1995 ▸; Wagner & Schönleber, 2009 ▸), order–disorder phenomena (Dornberger-Schiff, 1956 ▸) *etc*., once the twinning has been accounted for, completion of pseudo-merohedric twin structures is nowadays often no more difficult than non-twinned structures of similar complexity. Twin laws for twinning by (pseudo-)merohedry may be derived by coset decomposition of the crystal lattice point symmetry (Flack, 1987 ▸). However, in many cases, plausible twin operations can be obtained simply by inspection of the unit cell metrics. In less obvious cases, computer programs (*e.g.* Aroyo *et al.*, 2006 ▸; Boyle, 2014 ▸) have been written to derive twin laws using the algorithms described by Flack (1987 ▸). Details of structure solution itself, however, particularly tips and tricks for non-trivial cases, have received less attention. For many small-mol­ecule pseudo-merohedric twins in which twin component fractions are notably different, diffraction from the major component is often sufficiently dominant that structure solution is quite straightforward. This paper presents, by way of three worked examples of differing complexity, practical tips and hints for the more problematic case of crystals twinned by pseudo-merohedry in which the relative volume fractions of individuals are close to equal.

## General information   

**Figure 1:**
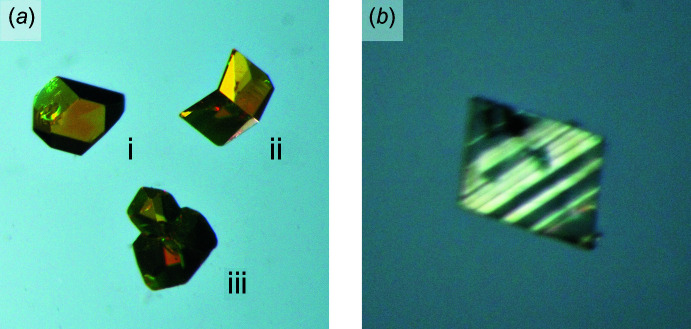
(*a*) Crystalline samples viewed between (partially) crossed polarizers of (i) a single crystal, (ii) a twin showing a re-entrant angle and different optical transmission on either side of a twin plane, and (iii) an aggregate, also showing re-entrant angles. The twin and aggregate could probably be cut with a razor blade to yield suitable single-crystal fragments. (*b*) An example of multiple domains within a two-individual twin for which microsurgery would be unlikely to yield a usable single-crystal fragment. Note: these images are representative examples from the author’s archives and are unrelated to the three cases discussed in depth in this paper.

**Figure 2:**
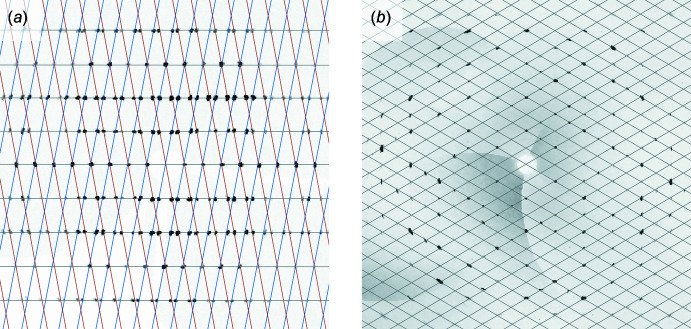
Reciprocal-lattice-slice reconstructions (*h*0*l*) for (*a*) a crystal twofold twinned by non-merohedry, requiring two orientation matrices to account for all diffraction maxima, and (*b*) a crystal twinned by pseudo-merohedry (see structure WUGLES, Section 4), for which a single orientation matrix accounts for all observed diffraction. The latter gives little outward indication of it being a twin, other than slight elongation of diffraction spots at higher 2θ angles.

Herbst-Irmer & Sheldrick (1998 ▸, 2002 ▸) and others (*e.g.* Rees, 1980 ▸; Yeates, 1988 ▸) describe a number of general observations and statistics that have proven useful for the identification and diagnosis of twinning. In addition, the failure of conventional direct methods of structure solution has also been noted as a common consequence of twinning (Parsons, 2003 ▸). Nowadays, most small-mol­ecule structures are solved by dual-space algorithms of one sort or another (*e.g.* Oszlányi & Sütő, 2004 ▸; Sheldrick, 2015*a* ▸). Such methods have proven immensely successful, such that their failure, persists as a strong indicator of twinning. Even prior to data collection, evidence of twinning is often apparent from optical microscopy (*e.g.* Fig. 1 ▸). Re-entrant angles (*e.g.* Kitamura *et al.*, 1979 ▸) between crystal faces, and optical extinction for transparent crystals viewed between crossed polarizers clearly indicate the presence of macroscopic twin domains (Hartshorne & Stuart, 1950 ▸; Nespolo & Ferraris, 2003 ▸ and references therein). Depending on the primary purpose of structure determination, where possible, it is often advisable to perform microsurgery to extricate a single-crystal fragment (Fig. 1 ▸ *a*), though this is not always feasible (Fig. 1 ▸ *b*). When modern auto-indexing routines [*e.g*. in *X-AREA* (Stoe & Cie, 2002 ▸); *CrysAlis PRO* (Rigaku OD, 2017 ▸); *APEX3* (Bruker, 2016 ▸)] return well-defined unit cells that only partially account for the observed diffraction maxima, twinning is often the culprit. Post data collection, reciprocal-lattice slice images a.k.a. ‘pseudo-precession pictures’ can readily expose twinning by non-merohedry, but are usually less useful for (pseudo-)merohedric twins (Fig. 2 ▸).

## Twofold pseudo-ortho­rhom­bic twinning, a straightforward example   

### Crystal and diffraction data assessment   

**Figure 3:**
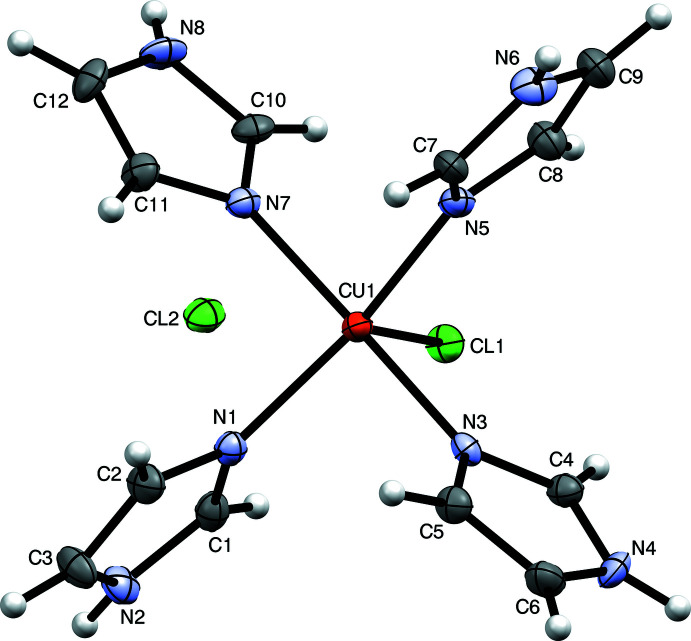
An ellipsoid plot of XUBNIR (50% probability), generated using *Mercury* (Macrae *et al.*, 2020 ▸).

Crystals of chloro­tetra­kis­(imidazole)­copper(II) chloride, C_12_H_16_Cl_2_CuN_8_, (Otieno *et al.*, 2001 ▸), XUBNIR in the CSD (Groom *et al.*, 2016 ▸), are monoclinic, with space group of type *P*2_1_/*n* and unit-cell parameters *a* = 8.8434 (2) Å, *b* = 13.2093 (4) Å, *c* = 13.8658 (5) Å, *β* = 90.0072 (18)°. Since the *β* angle is so close to 90°, the unit cell is metrically ortho­rhom­bic, even though the underlying symmetry is monoclinic. This situation corresponds to criterion (*a*) in the list of classic symptoms of twinning outlined by Herbst-Irmer & Sheldrick (1998 ▸). The mol­ecular structure of XUBNIR, which consists of a square-based pyramidal Cu^II^ with four N-bound imidazoles, a bound chlorine ligand and one free chloride anion (Fig. 3 ▸), seems innocuous. Data collection and processing for XUBNIR were also unremarkable. To allow the reader to follow along, a dataset is available in the supporting information.

Under the initial assumption that this is a routine structure, *XPREP* (Sheldrick, 2008 ▸) was used to set up files for structure solution. Not surprisingly, the program suggests a primitive ortho­rhom­bic unit cell, which has a seemingly respectable *R*_sym_ of 4.2% (Table 1 ▸ *a*). Analysis of systematic absences, however, does not lead to an acceptable ortho­rhom­bic space group (Table 1 ▸ *b*). Impossible systematic absences are another classic symptom of twinning, corresponding to criterion (*e*) described by Herbst-Irmer & Sheldrick (1998 ▸). *XPREP* does suggest 2_1_ screw axes associated with each of *a*, *b*, and *c*, as well as an *n*-glide plane perpendicular to *b*. Thus, given the information at hand, even in the absence of any particular knowledge of the suspected twinning, the obvious way forward is to consider the next lowest symmetry crystal system, monoclinic. In *XPREP*, this requires overriding the default crystal system suggestion (Table 1 ▸ *c*), upon which the program suggests a space group of type *P*2_1_/*n*, with *R*_sym_ = 2.3%. Other potential monoclinic settings can be sidelined as possibilities at this point because they are not consistent with the *n*-glide and they each give a worse *R*_sym_, similar to that of the rejected ortho­rhom­bic cell.

### Twin operations by inspection of unit-cell parameters for XUBNIR   

**Figure 4:**
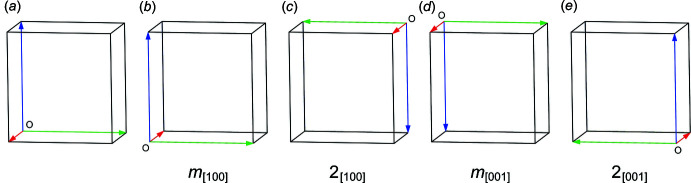
(*a*) Unit-cell axes for XUBNIR (red = *a*, green = *b*, blue = *c*), and after transformation by: (*b*) a mirror plane perpendicular to the *a* axis, (*c*) a twofold rotation about the *a* axis, (*d*) a mirror plane perpendicular to the *c* axis, (*e*) a twofold rotation about the *c* axis. All five of the unit-cell ‘boxes’ superimpose because *β* ≃ 90°. Note that the mirror operations change the right-handed coordinate system to left handed, whereas the twofold rotations preserve the handedness. Diagrams generated using *Mercury* (Macrae *et al.*, 2020 ▸).

The essence of twinning in this structure lies in the difference between ortho­rhom­bic and monoclinic symmetry. For a primitive monoclinic crystal, there could be twofold rotation, 2_1_ screw, mirror, *a* or *c*-axial, or *n*-diagonal glide planes associated only with the *b* axis (assuming the monoclinic *b*-unique convention is respected). For ortho­rhom­bic crystals, these symmetry elements may each be associated with *a*, *b*, and *c*. In reciprocal space, the translational parts of screw and glide operations manifest only as systematic absences, so in the context of twinning, we need only consider the point-symmetry operations rotation, reflection, and inversion (*vide supra*, Section 1). For consideration as twin operations in XUBNIR, that limits the analysis to mirror and twofold rotation operations associated with the *a* and *c* axes. Such mirror operations change the sign of just one index, while twofold rotations flip the sign of two indices. The feasible twin operations, expressed as (3×3) transformation matrices, are thus:
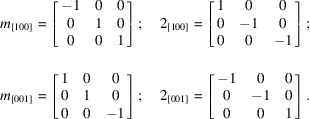



Matrices *m*_[100]_ and *m*_[001]_ describe reflection across mirror planes perpendicular to *a* and *c* while 2_[100]_ and 2_[001]_ describe 180° (*i.e.* twofold) rotation about *a* and *c*, respectively. The effect of these operations on the unit-cell axes are shown in Fig. 4 ▸ *a*–*e*. Since the structure is centrosymmetric, *m*_[100]_ and 2_[100]_ are equivalent for this unit cell, as are *m*_[001]_ and 2_[001]_. Similarly, since monoclinic symmetry has either *m*, 2 or 2/*m* point symmetry (by convention associated with the *b* axis), the sign of *b* (*i.e.* mirror plane perpendicular to *b*) or signs of *a* and *c* (*i.e.* twofold rotation about *b*) can be flipped. Thus, in reciprocal space for this structure, *m*_[100]_, 2_[100]_ *m*_[001]_, 2_[001]_, all produce the same effect when used as twin matrices to transform reflection indices, and thereby constitute the twin law.

### Structure solution   

The structure of XUBNIR does not solve with the correct space group when using the iterative dual-space method in *SHELXT* (Sheldrick, 2015*a* ▸) or by charge-flipping (Oszlányi & Sütő, 2004 ▸) as implemented in *PLATON* (Spek, 2020 ▸). Recognizable, albeit rudimentary but ultimately usable solutions are, however, possible using the conventional direct methods programs *SHELXS* (Sheldrick, 2008 ▸) and *SIR* (Altomare *et al.*, 1999 ▸), and possibly other programs not directly intended for twins. Nevertheless, the dual-space recycling algorithm used in *SHELXD* (Sheldrick, 2008 ▸) can include two twin components in a straightforward way, and results in a starting model that is quite easy to complete. The following instructions file for *SHELXD* was generated using *XPREP*, but has been hand edited to include twin matrix 2_[001]_.

TITL XUBNIR in P2(1)/n

CELL 0.71073 8.8434 13.2093 13.8658 90.0000 90.0072 90.0000

ZERR 4.00 0.0002 0.0004 0.0005 0.0000 0.0018 0.0000

LATT 1

SYMM 0.5-X, 0.5+Y, 0.5-Z

SFAC C H N CL CU

UNIT 64 64 16 8 4

TWIN -1 0 0 0 -1 0 0 0 1

FIND 15

PLOP 20 25 28

MIND 1.0 -0.1

NTRY 1000

HKLF 4

END

**Figure 5:**
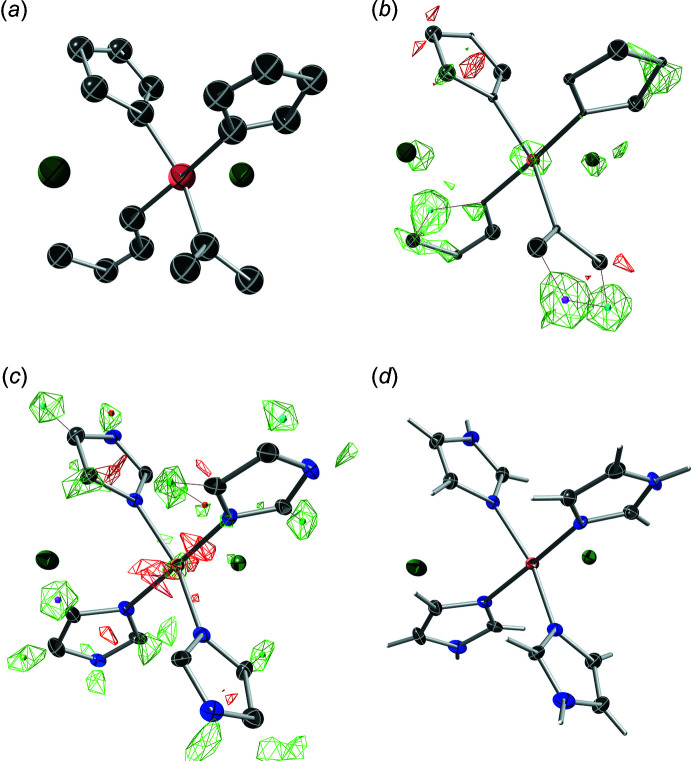
Snapshots of model building and refinement of XUBNIR. (*a*) Initial structure solution from *SHELXD*. (*b*) After isotropic refinement, three missing atoms are clearly visible as difference-map peaks, and the relative size of displacement spheres allow most carbon and nitro­gen atoms to be distinguished. (*c*) After anisotropic refinement, all hydrogen atoms are clearly present in a difference map. (*d*) After inclusion of hydrogen atoms. Diagrams were generated using *ShelXle* (Hübschle *et al.*, 2011 ▸).

In the above, 1000 trials (command NTRY) are overkill, but the structure is small so it runs quite quickly on modern computers. The default for twin component volume fractions is 0.5, but can be changed by a BASF parameter, as per *SHELXL* (Sheldrick, 2015*b* ▸). The resulting preliminary model (Fig. 5 ▸ *a*) is fairly complete. It is missing only three atoms and has the imidazole nitro­gen atoms mis-assigned as carbon; all problems that are easily fixed. A few cycles of model building and refinement (Fig. 5 ▸ *b*–*d*) proves to be no more complicated than for a routine (non-twinned) single-crystal structure. The final model has refined twin component volumes of about 54% and 46% and an *R*_1_ value of 2.5%

## Threefold pseudo-hexa­gonal twinning using a non-conventional space group setting, *B*2_1_   

**Figure 6:**
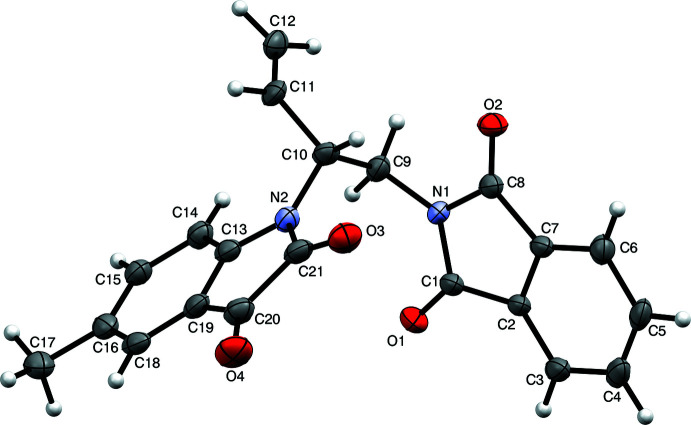
An ellipsoid plot (50% probability) of one representative mol­ecule of WUGLES (out of six independent mol­ecules in the asymmetric unit), generated using *Mercury* (Macrae *et al.*, 2020 ▸).

Crystals of the chiral compound 1-{(*R*)-1-[(3-oxo-2-iso­indolino­yl)meth­yl]-2-propen­yl}-5-methyl-2,3-indolinedione, C_21_H_16_N_2_O_4_, Fig. 6 ▸, (Trost *et al.*, 2020 ▸), WUGLES in the CSD (Groom *et al.*, 2016 ▸), form as orange elongated hexa­gonal columnar needles. Initial indexing of the diffraction pattern gives a unit cell that appears to be primitive hexa­gonal. All attempts to solve the structure using hexa­gonal or trigonal symmetry, however, failed; reminiscent of criterion (*d*) described by Herbst-Irmer & Sheldrick (1998 ▸). A chemically reasonable structure was eventually found that had twelve mol­ecules in the asymmetric unit of a space group of type *P*2_1_. Refinement of this *Z*′ = 12 model as a threefold twin seemed promising, but became unstable when displacement parameters were made anisotropic. Subsequent analysis showed that for the pseudo-hexa­gonal setting, the individuals had to be *B*-centred with an asymmetric unit having *Z*′ = 6, and threefold twinned, requiring the unconventional space group (see, for example, Nespolo & Aroyo, 2016 ▸) setting *B*2_1_, and similar twin component volumes. A thorough description of the twinning in WUGLES was subsequently given by Nespolo *et al.* (2020 ▸). Detailed steps involved in structure solution, are given here. The full dataset is available in the supporting information.

### Diffraction data analysis for WUGLES using *XPREP*   

On reading the diffraction data into *XPREP*, the program suggests two plausible primitive unit cells, one hexa­gonal with *R*_sym_ = 7.8% and one monoclinic having *R*_sym_ = 2.0% (Table 2 ▸ *a*). It also suggests five *C*-centred ortho­rhom­bic and monoclinic unit cells, but these can be rejected immediately due to their unacceptable *R*_sym_ values of about 30% or so. All attempts to solve the structure using hexa­gonal or trigonal symmetry failed miserably. Indeed, since the *R*_sym_ for hexa­gonal is almost four times that of primitive monoclinic, *XPREP* suggests the latter as its default. The next task is to assign a tentative space group. Systematic absences (Table 2 ▸ *b*) indicate a 2_1_ screw axis parallel to *b*. Since the compound was known from the synthesis to be chiral and enanti­opure, the suggestion of *P*2_1_/*m* can be rejected, leaving only *P*2_1_. Without additional information, this is the best we can do at this stage.

### Suspected threefold twinning and a plausible twin law for WUGLES   

**Figure 7:**
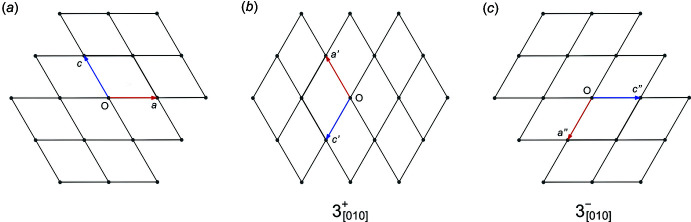
(*a*) A stack of (primitive) unit cells with highlighted axes for WUGLES viewed in projection down the *b* axis, and after rotation by: (*b*) 120° and (*c*) 240° (eq. −120°) about *b*. Diagrams generated using *XP* in *SHELXTL* (Sheldrick, 2008 ▸).

The unit-cell parameters for the as-indexed monoclinic *P* setting have *a* ≃ *c* and *β* ≃120°, so threefold pseudo-merohedric twinning about the *b* axis is a reasonable supposition and is consistent with criterion (*d*) of Herbst-Irmer & Sheldrick (1998 ▸). A threefold rotation requires successive rotational increments of 120°, with the third step reproducing the starting position. For positive rotation (anti­clockwise) about *b*, this corresponds to the following matrices:
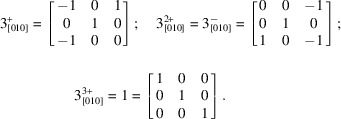



The transformational effects of these matrices on the unit-cell axes are illustrated in Fig. 7 ▸.

### Initial structure solution for WUGLES using *P*2_1_   

A single mol­ecule of WUGLES has 27 non-hydrogen atoms. Given the relatively large volume of the primitive monoclinic cell [*V* = 10357.0 (9) Å^3^], for *P*2_1_ to be correct, the asymmetric unit for the pseudo-hexa­gonal cell could accommodate twelve mol­ecules, *i.e.* 234 (C, N, O) atoms. Perhaps not surprisingly in view of the expected twinning and large number of similar sized atoms, neither conventional direct methods nor iterative dual-space algorithms (*SHELXT* or charge flipping in *PLATON*) are able to readily solve this structure. The dual-space recycling algorithm in *SHELXD*, however, is able to provide a starting model with recognizable chemical fragments. Although the current version of *SHELXD* (v2013/2) is restricted to just two twin components (rather than the suspected three in WUGLES), its success rate is higher if two twin components of equal volume are included (rather than just a single component), *i.e.* using the transpose of either the 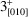 or 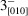 matrices defined above. A further increase in the success rate is possible *via* random omission of some fraction of the atoms during the dual-space recycling using the command ‘WEED’ in *SHELXD*, a form of rand­om­ized ‘omit’ map (Bhat & Cohen, 1984 ▸), which has the effect of reducing phase bias for non-centrosymmetric structures. Thus, in the following input file for *SHELXD* written by *XPREP*, the added TWIN and WEED commands enable a dramatic reduction in the number of trials (command NTRY) from the default 1000 to about 20.

TITL WUGLES in P2(1)

CELL 1.54178 15.5993 49.062 15.6099 90.000 119.896 90.000

ZERR 24.00 0.0008 0.002 0.0008 0.000 0.002 0.000

LATT -1

SYMM -X, 0.5+Y, -Z

SFAC C H N O

UNIT 504 384 48 96

FIND 194

PLOP 259 324 362

MIND 1.0 -0.1

TWIN 0 0 1 0 1 0 -1 0 -1

NTRY 20

WEED 0.3

HKLF 4

END

**Figure 8:**
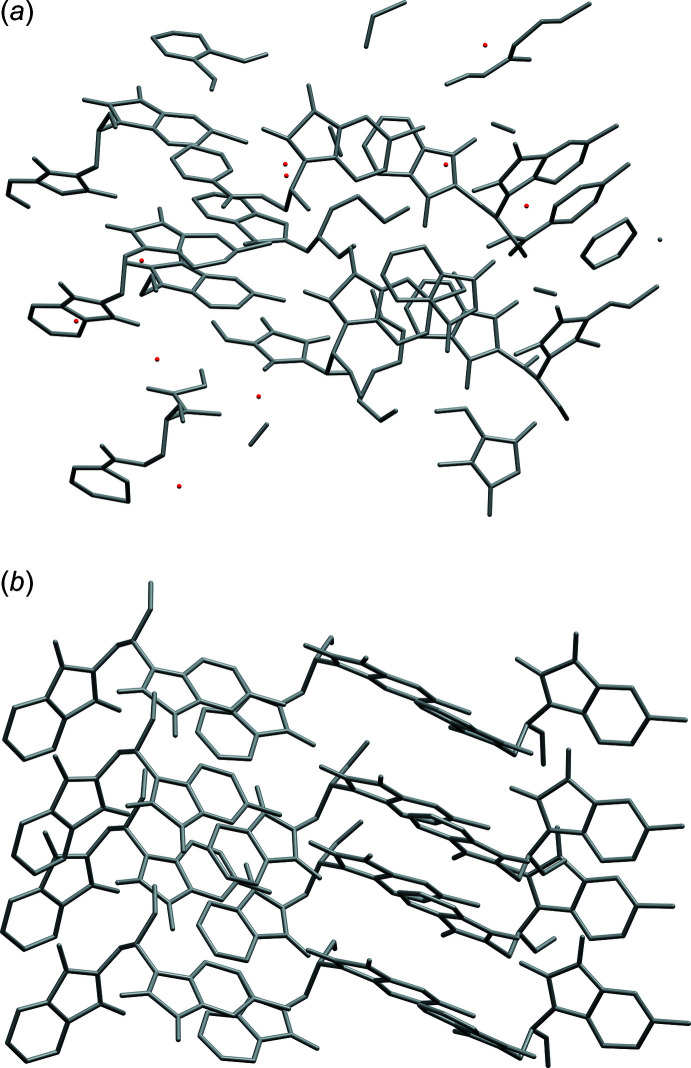
(a) Initial *P*2_1_, *Z*′ = 12 structure solution for WUGLES. (*b*) *P*2_1_, *Z*′ = 12 structure after model building and isotropic refinement. Diagrams generated using *Mercury* (Macrae *et al.*, 2020 ▸).

The resulting structure solution prior to any model building and refinement is shown in Fig. 8 ▸ *a*. This model is far from complete, but aside from a few disconnected parts, there are many recognizable mol­ecular fragments, including the expected six- and five-membered rings. A few rounds of model building and isotropic refinement with threefold twinning included rapidly generates the whole asymmetric unit for the *P*2_1_ cell (Fig. 8 ▸ *b*), resulting in an *R*_1_ value of about 9.5%. Under normal circumstances, the next step would be to complete the structure by including anisotropic displacement parameters (ADPs) and adding hydrogen atoms. However, all attempts to refine ADPs for this model were wildly unstable. A search for missed symmetry using *ADDSYM* in *PLATON*, however, proved to be fruitless. Even with inclusion of an extensive battery of restraints, there were still hefty correlations between pairs of similar-geometry mol­ecules in the least-squares refinement. At this stage, therefore, it seemed likely that the actual asymmetric unit of each individual was only half as large as required by the current *P*2_1_ model (*i.e. Z*′ ought to be 6, not 12). Possible causes therefore included each component having a primitive cell with either *a* or *c* (but not both) only half as long, or alternatively, *B*-centring. Given the threefold twinning about *b*, each scenario would result in a diffraction pattern for the twin that is indexable as *apparently* primitive and pseudo-hexa­gonal. To investigate further, the ‘LIST 8’ command in *SHELXL* was used to generate ‘detwinned’ data. The resulting *SHELXL* format *fcf* file contains the following information for each reflection: *h*, *k*, *l*, 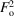, 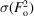, 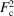, 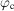, *d*, 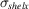. The first five fields may be easily converted to an ‘HKLF 4’ format data file using the *unix* (*Linux*, *MacOS*, etc.) utility *awk* [also available for *Windows via* the *Cygwin* project (Cygwin, 2020 ▸)], as follows:

awk ’{printf "%4d%4d%4d%8.2f%8.2f\n", $1,$2,$3,$4,$5}’ in.fcf > out.hkl

where *in.fcf* and *out.hkl* are the input ‘LIST 8’ format *fcf* file (after removal of its CIF format header) and output ‘HKLF 4’ format *hkl* files, respectively. Comparison of intensities for the full dataset with this ‘detwinned’ data (Table 3 ▸ *a*,*b*) clearly show that for this unit-cell setting, the individual is *B*-centred. Thus, the pseudo-hexa­gonal unit cell, originally indexed as primitive using reflections from the whole three-component twin, actually corresponds to threefold rotational twinning of *B*-centred cells of the individuals, each requiring an unconventional space group, *B*2_1_. Thus, twinning in WUGLES is by reticular pseudo-merohedry with zero obliquity (the twin axis is coincident with the unit-cell *b* axis), but non-zero twin misfit (since *β* is not exactly 120°, twin-related lattice points do not exactly superimpose). A rigorous analysis is given by Nespolo *et al.* (2020 ▸). This *B*-centred cell may, of course, be transformed to a conventional primitive cell with half the volume. However, such a smaller *P*2_1_ cell is pseudo-ortho­rhom­bic, and thus the threefold nature of the twinning becomes far less intuitive than for the larger pseudo-hexa­gonal cell. Moreover, refinement using *SHELXL* would then require conversion of the ‘HKLF 4’ format twinned datafile to a much larger ‘HKLF 5’ format dataset. That is possible (*e.g.* by adaptation of the scheme in Appendix A[App appa]), but for WUGLES, use of the unconventional *B*-centred setting is far more elegant, at least for refinement using *SHELXL*. Technical details of the equivalence of the *B*2_1_ versus *P*2_1_ description are described at length in Nespolo *et al.* (2020 ▸).

### Structure solution and refinement for WUGLES using *B*2_1_   

**Figure 9:**
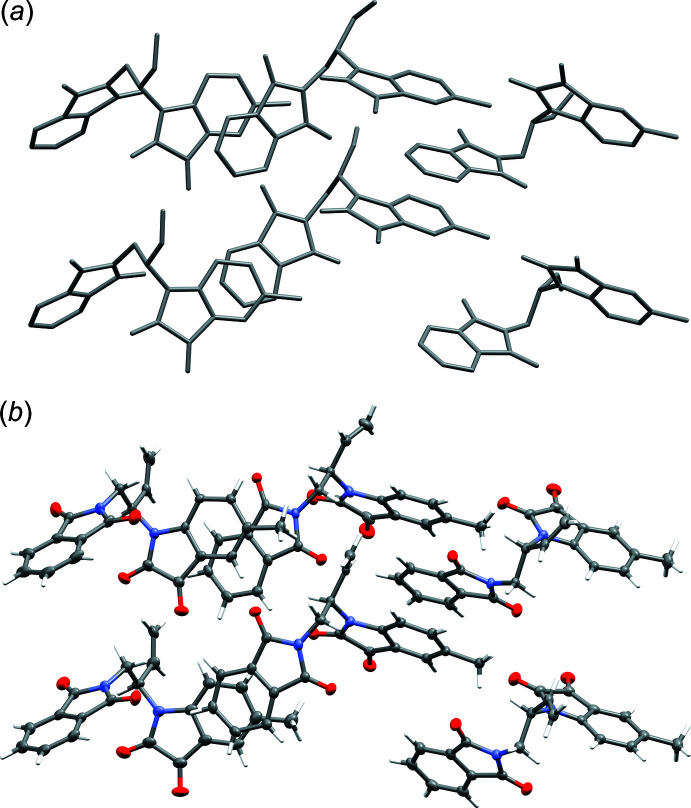
(*a*) Structure solution using *B*2_1_, *Z*′ = 6 for WUGLES, all non-hydrogen atoms are present. (*b*) The *B*2_1_, *Z*′ = 6 structure with hydrogen atoms and after full anisotropic refinement and assignment of absolute configuration. Diagrams generated using *Mercury* (Macrae *et al.*, 2020 ▸).

The initial (subsequently shown to be incorrect and unref­inable) *P*2_1_, *Z*′ = 12 model in the larger primitive pseudo-hexa­gonal unit cell could quite easily be pared down by removing one member of each symmetry-equivalent pair of mol­ecules. Nonetheless, given the speed of modern computers, it is perhaps easier to simply re-solve the structure using the *B*2_1_ setting. For *SHELXD* this requires one trivial edit to the instructions file, namely, changing the LATT command from ‘LATT -1’ (primitive non-centrosymmetric) to ‘LATT -6’ (*B*-centred non-centrosymmetric). Using the previously obtained detwinned data and the symmetry of *B*2_1_, *SHELXD* easily finds all the non-hydrogen atoms (Fig. 9 ▸ *a*). In spite of the circuitous route taken to *solve* the structure, subsequent *refinement* carried out against the full dataset with threefold twinning included proceeds smoothly for the *B*2_1_ model. It requires no constraints or restraints, even for a fully anisotropic model, with all hydrogen atoms having been found in difference-Fourier maps and included in the refinement (Fig. 9 ▸ *b*). The absolute configuration could also be determined from the diffraction data using established methods (Flack, 1983 ▸; Hooft *et al.*, 2008 ▸; Parsons *et al.*, 2013 ▸).

## Fourfold pseudo-tetra­gonal twinning *via* an *I*-centred supercell   

**Figure 10:**
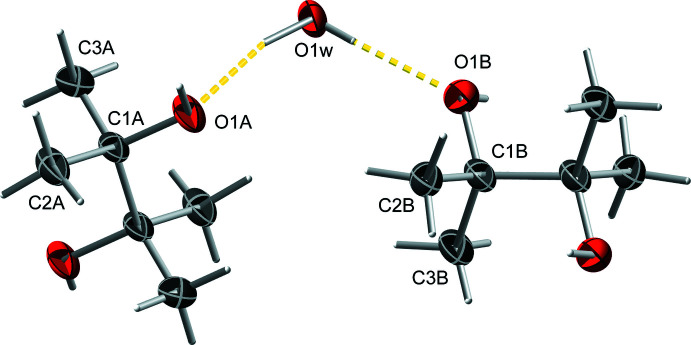
An ellipsoid plot (50% probability) of SAXDUR, generated using *ShelXle* (Hübschle *et al.*, 2011 ▸). The simplicity of the mol­ecule belies the complexity of the twinning.

Not all cases of twinning by pseudo-merohedry can ultimately be accounted for using the TWIN command in *SHELXL*. The crystal structure of pinacol monohydrate has primitive monoclinic symmetry of type *P*2/*n* (space group 13), but is fourfold twinned by virtue of a pseudo-tetra­gonal *I*-centred supercell. The monohydrate phase of crystalline pinacol was identified by Pushin & Glagoleva (1922 ▸), but its structure (Fig. 10 ▸) remained unsolved until 2003 (Hao *et al.*, 2005 ▸; SAXDUR in the CSD).

### Diffraction pattern indexing and data analysis for SAXDUR   

The crystal used for SAXDUR initially indexed to give cell parameters *a* = 12.9001 (7) Å, *b* = 12.8941 (7) Å, *c* = 12.8917 (9) Å, α = 107.517 (3)°, β = 110.359 (3)°, γ = 110.581 (3)°. This triclinic setting was used for data collection to ensure that no experimental information was inadvertently lost or skipped, but without making any assumptions about crystal symmetry. The resulting dataset is available in the supporting information. The similarity of the above three axis lengths and of the β and γ angles, however, immediately portend transformation to a higher symmetry cell. A search for higher symmetry using *XPREP* returned eight possible centred cells; one tetra­gonal-*I*, one ortho­rhom­bic-*I*, three monoclinic-*I*, and their three monoclinic-*C* equivalents (which were dismissed as they have β ≃ 134°, thereby obscuring the pseudo-tetra­gonal symmetry). The *I*-centred cases are reproduced in Table 4 ▸. The tetra­gonal-*I* cell (option A) was dismissed due to its much higher *R*_sym_ and because systematic absences were inconsistent with any tetra­gonal space group [criterion (*e*) of Herbst-Irmer & Sheldrick, 1998 ▸]. Moreover, the crystal itself did not exhibit optical extinction characteristic of tetra­gonal symmetry (Hao *et al.*, 2005 ▸). For ortho­rhom­bic-*I*, *XPREP* suggests a space group of type *Ibca* (Table 5 ▸ *a*), but this also proved to be a dead end. All attempts to find a chemically reasonable structure for ortho­rhom­bic-*I* failed, with or without consideration of twinning. This leaves the three monoclinic-*I* settings (options D, F, H in Table 4 ▸), each having similar *R*_sym_ and cell angles all ∼90°. As a worst-case scenario, all three settings would need to be considered, with only the right one expected to yield a viable structure model. It makes sense to first consider the setting that gives the lowest *R*_sym_ (option F); with hindsight it also happens to be the correct choice. This cell has the same transformation matrix (from the initial primitive cell) as the ortho­rhom­bic-*I* option and has systematic absences consistent with space groups of type *I*2/*a* and *Ia* (Table 5 ▸ *b*).

### Suspected twinning for SAXDUR   

The unit-cell metrics of the chosen *I*-centred monoclinic cell are consistent with pseudo-tetra­gonal four-component twinning about its *c* axis. For positive rotation, four successive 90° steps about *c* are required, yielding the following four matrices:
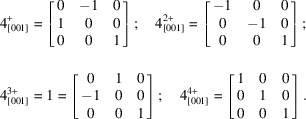



Two 90° steps generate a twofold rotation (as for a pseudo-ortho­rhom­bic twin), while the fourth step regenerates the starting position. The presence of pseudo-tetra­gonal twinning would result in significant populations for four individuals, as opposed to two if it were a pseudo-ortho­rhom­bic twin.

### Initial structure ‘solution’ for SAXDUR using *I*2/*a*   

The following instructions file for *SHELXD* created by *XPREP* has been edited to include matrix 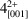, but 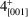 or 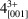 could also be tried.

TITL monI in I2/a

CELL 0.71073 14.6876 14.7273 15.2443 90.0000 90.027 90.0000

ZERR 16.00 0.0010 0.0011 0.0011 0.0000 0.005 0.0000

LATT 2

SYMM 0.5-X, Y, -Z

SFAC C H O

UNIT 96 256 48

TWIN -1 0 0 0 -1 0 0 0 1

FIND 15

PLOP 21 26 29

MIND 1.0 -0.1

NTRY 1000

HKLF 4

END

**Figure 11:**
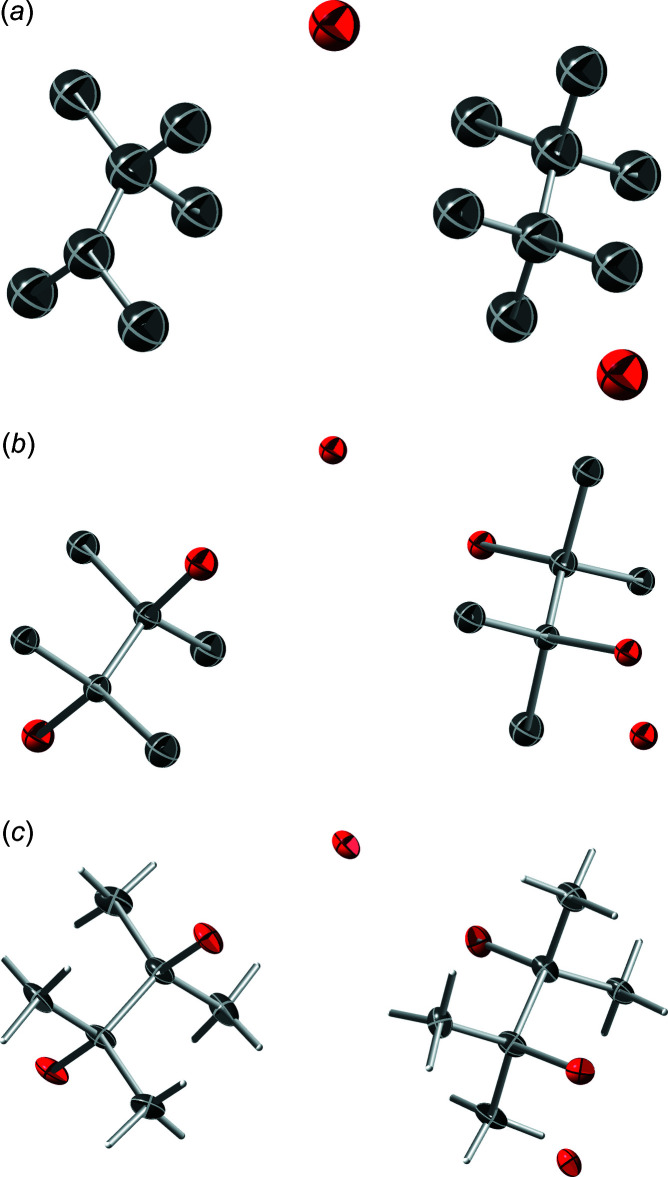
Preliminary structure for SAXDUR using the *I*-centred supercell: (*a*) Initial *SHELXD* solution (using two twin components). (*b*) After model building and isotropic refinement. (*c*) With anisotropic displacement parameters and riding methyl hydrogens (four twin components). Diagrams generated using *ShelXle* (Hübschle *et al.*, 2011 ▸).

Since the structure is quite small, even a thousand trials (‘NTRY 1000’) runs quickly. On completion of the *SHELXD* run, the resulting model is not complete (Fig. 11 ▸ *a*), but shows enough of the structure to easily build two pinacol mol­ecules and assign two water oxygens (Fig. 11 ▸ *b*). Fourfold twinning can then be tested by fourfold application of either twin matrix 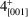 or 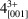, for example, by including (for the former) the following commands in an *ins* file for *SHELXL*.

TWIN 0 -1 0 1 0 0 0 0 1 4

BASF 0.25 0.25 0.25

The component fractions in the above *SHELXL* ‘BASF’ command are just initial guesses and will refine. After a few cycles of least-squares refinement, the model is dramatically improved. Even anisotropic refinement (with restraints) is possible, as is addition of riding methyl hydrogens (Fig. 11 ▸ *c*). All of the refined BASF parameters are significant, indicating that fourfold twinning is appropriate. Consequently, the *R*-value drops well into single digits.

### Search for missed symmetry   

**Figure 12:**
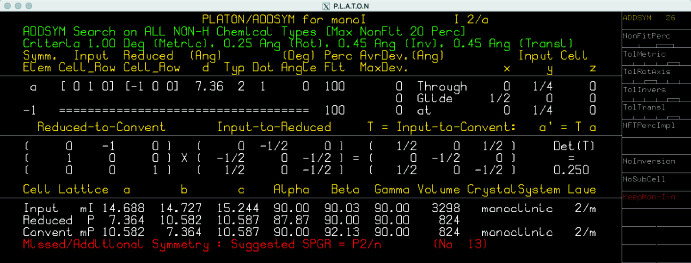
A search for missed symmetry using *ADDSYM* in *PLATON* (Spek, 2020 ▸) reveals that the symmetry of untwinned individuals in SAXDUR is actually *P*2/*n* rather than *I*2/*a*. Thus, the true asymmetric unit contains only half as many atoms as in the *I*2/*a* model.

In spite of the progress, the current *I*2/*a* model has demonstrable problems. A careful inspection reveals hefty correlation between atoms related through the central bond of each pinacol mol­ecule, suggestive of missed inversion symmetry. Thus, a careful check for missing symmetry, visually and for example, using *ADDSYM* in *PLATON* (Spek, 2020 ▸) is warranted. For the latter, *PLATON* requires a *CIF* and a ‘LIST 4’ format *fcf* file, which are written by *SHELXL* if both ‘ACTA’ and ‘LIST 4’ commands are specified in the *SHELXL ins* file. *ADDSYM* predicts a primitive monoclinic (*P*2/*n*) cell with a volume only a quarter as large as the current *I*-centred cell (Fig. 12 ▸), and supplies a transformation matrix from the *I*2/*a* setting to *P*2/*n*, namely:
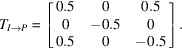



It also gives the option to save a copy of the transformed model (option *ADDSYM-SHX*).

**Figure 13:**
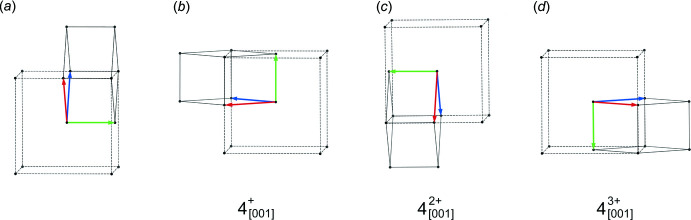
(*a*) Relationship between the pseudo-tetra­gonal *I*-centred supercell (dashed lines) and a primitive monoclinic individual (solid black lines, plus red = *a*, green = *b*, blue = *c*) of the twin in SAXDUR, viewed slightly off the fourfold twin axis, and after anti­clockwise rotations of (*b*) 90°, (*c*) 180°, and (*d*) 270° about the twin axis. Diagrams generated using *VESTA* (Momma & Izumi, 2011 ▸).

Since the volume of the *P*2/*n* cell is only a quarter the size of the *I*2/*a* cell, its asymmetric unit is half as big, which means the twin index for SAXDUR is 2. Thus, the twinning is reticular; each individual only contributes to half of the measured diffraction maxima of the twin. Transformation of the dataset for *P*2/*n* by successive 90° rotations about the fourfold twin axis (Fig. 13 ▸) would therefore generate non-integer reflection indices for half the data. Normally, that is not a problem as they’d be simply ignored or deleted; non-integer indices do not represent actual Bragg peaks. Nevertheless, it causes such ‘impossible’ indices to coincide with actual Bragg maxima from other individuals. Similar to WUGLES, we should not simply discard them, but for SAXDUR, there is no setting of space group 13, conventional or otherwise, that would allow use of the *SHELXL* ‘TWIN’ command. The way forward is to make a data file in *SHELXL* ‘HKLF 5’ format that preserves all the information. One approach to creation of such an ‘HKLF 5’ format datafile for SAXDUR is given in a series of straightforward steps in Appendix A[App appa]. The resulting datafile is available in the supporting information.

### Complete refinement of SAXDUR as a fourfold twin using *P*2/*n*   

**Figure 14:**
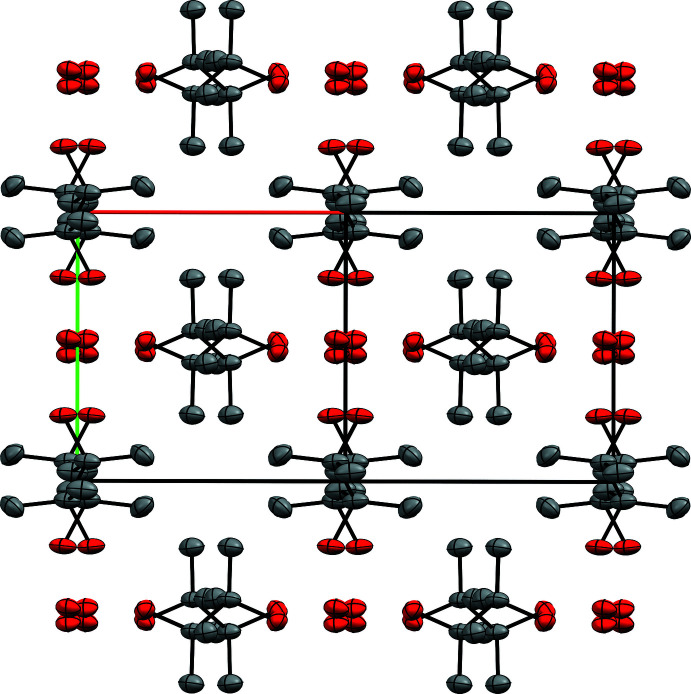
The propensity for pseudo-tetra­gonal fourfold twinning in SAXDUR is clearly demonstrated in a packing plot for one individual viewed along [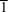
01], which is the fourfold twin axis relative to the *P*2/*n* unit cell. Diagram generated using *Mercury* (Macrae *et al.*, 2020 ▸).

After minor editing, the *P*2/*n* model supplied by *ADDSYM* in *PLATON* refines smoothly as a four-component twin against the ‘HKLF 5’ format dataset without the need for restraints (Fig. 10 ▸). When viewed down the [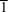
01] direction of the *P*2/*n* cell, which corresponds to the pseudo-tetra­gonal *c* axis of the *I*2/*a* supercell, the approximate fourfold symmetry is readily apparent (Fig. 14 ▸).

## Conclusions   

Any ultimately correct structure determination of a twinned crystal requires use of the proper space-group symmetry and complete treatment of the twinning. Once these criteria are met, final refinement of the structure is usually no more problematic than a non-twinned structure of similar complexity. Nevertheless, the route taken to solve the structure and assign the true space-group symmetry and twin law might in practice be rather indirect. As such, whatever tools and tricks are used as means to delivering a valid crystallographic end result are fair game.

## Supplementary Material

Structure factors: contains datablock(s) . DOI: 10.1107/S205698902100342X/hb7973sup1.hkl

Input file for structure solution of XUBNIR by SHELXD. DOI: 10.1107/S205698902100342X/hb7973sup2.ins.txt

Structure factors: contains datablock(s) . DOI: 10.1107/S205698902100342X/hb7973sup3.hkl

Input file for P21 supercell structure solution of WUGLES by SHELXD. DOI: 10.1107/S205698902100342X/hb7973sup4.ins.txt

Structure factors: contains datablock(s) . DOI: 10.1107/S205698902100342X/hb7973sup5.hkl

Structure factors: contains datablock(s) . DOI: 10.1107/S205698902100342X/hb7973sup6.hkl

Input file for I2/a supercell solution of WUGLES by SHELXD. DOI: 10.1107/S205698902100342X/hb7973sup7.ins.txt

Structure factors: contains datablock(s) . DOI: 10.1107/S205698902100342X/hb7973sup8.hkl

Steps for generation of an HKLF 5 format datafile from an HKLF 4 format datafile for structure SAXDUR (pinacol monohydrate), in plain ASCII text suitable for copy/paste. DOI: 10.1107/S205698902100342X/hb7973sup9.txt

Additional supporting information:  crystallographic information; 3D view; checkCIF report

## Figures and Tables

**Table:** 

(*a*) Search for higher metric symmetry:	
Option A:	FOM = 0.007 deg. ORTHORHOMBIC P-lattice **R(sym) = 0.042** [4007]
Cell:	8.843 13.209 13.866 90.00 90.01 90.00 Volume: 1619.74
Matrix:	1.0000 0.0000 0.0000 0.0000 1.0000 0.0000 0.0000 0.0000 1.0000

**Table:** 

(*b*) Space group assignment fails for orthorhombic:												
Systematic absence exceptions (for ortho­rhom­bic P):												
	b--	c--	n--	**21--**	-c-	-a-	**-n-**	**-21-**	--a	--b	--n	**--21**
N	596	595	595	20	366	371	373	29	423	425	424	24
N I>3s	408	464	386	0	179	180	1	0	364	343	315	0
<I>	41.1	24.4	42.4	**0.1**	39.7	39.1	**0.2**	**0.2**	59.7	60.8	35.0	**0.1**
<I/s>	11.7	13.1	11.3	**0.6**	10.8	10.7	**0.5**	**0.6**	15.3	14.5	12.0	**0.4**
**No acceptable space group** - change tolerances or unset chiral flag or possibly change input lattice type, then recheck cell using H-option												

**Table:** 

(*c*) Space group assignment is successful for monoclinic:									
Systematic absence exceptions (default overridden to monoclinic):									
	**-21-**	-a-	-c-	**-n-**					
N	29	371	366	373					
N I>3s	0	180	179	1					
<I>	**0.2**	39.1	39.7	**0.2**					
<I/s>	**0.6**	10.7	10.8	**0.5**					
Identical indices and Friedel opposites combined before calculating R(sym)									
Option	Space Group	No.	Type	Axes	CSD	**R(sym)**	N(eq)	Syst. ABS.	CFOM
[A]	**P2(1)/n**	#14	centro	1	19410	**0.023**	2646	0.6 / 10.7	4.28

**Table:** 

(*a*) Search for higher metric symmetry:	
Option A:	FOM = 0.126 deg. HEXAGONAL P-lattice **R(sym) = 0.078** [28182]
Cell:	15.599 15.610 49.062 90.00 90.00 119.90 Volume: 10357.03
Matrix:	1.0000 0.0000 0.0000 0.0000 0.0000 1.0000 0.0000 -1.0000 0.0000
	
Option E:	FOM = 0.000 deg. MONOCLINIC P-lattice **R(sym) = 0.020** [20598]
Cell:	15.599 49.062 15.610 90.00 119.90 90.00 Volume: 10357.03
Matrix:	1.0000 0.0000 0.0000 0.0000 1.0000 0.0000 0.0000 0.0000 1.0000

**Table:** 

(*b*) Space group assignment for primitive monoclinic:									
Systematic absence exceptions (for monoclinic P):									
	**-21-**	-a-	-c-	-n-					
N	249	2180	2178	2180					
N I>3s	2	1136	1066	1208					
<I>	**0.1**	2.8	2.4	3.0					
<I/s>	**0.6**	4.8	4.4	5.1					
Identical indices and Friedel opposites combined before calculating R(sym)									
Option	Space Group	No.	Type	Axes	CSD	R(sym)	N(eq)	Syst. ABS.	CFOM
[A]	**P2(1)**	#4	chiral	1	3543	0.020	20598	0.6 / 4.4	4.41
[B]	P2(1)/m	#11	centro	1	402	0.020	20598	0.6 / 4.4	3.85

**Table:** 

(*a*) Diffraction intensities for the full twinned dataset of WUGLES imply a primitive lattice.									
Lattice exceptions:	P	A	**B**	C	I	F	Obv	Rev	All
N (total) =	0	174957	175199	174948	174780	262552	233389	233137	349710
N (int>3sigma) =	0	132735	128777	132894	132671	197203	177681	177307	266364
Mean intensity =	0.0	7.9	**3.8**	7.9	7.9	6.5	7.8	7.9	7.9
Mean int/sigma =	0.0	7.2	**6.4**	7.2	7.2	7.0	7.2	7.3	7.3
**Lattice type: P chosen** Volume:10357.03									
									

**Table:** 

(*b*) Diffraction intensities for ‘detwinned’ data of WUGLES confirm *B*-centring.									
Lattice exceptions:	P	A	**B**	C	I	F	Obv	Rev	All
N (total) =	0	21799	21879	21794	21786	32736	29121	29125	43679
N (int>3sigma) =	0	10893	2114	10881	10938	11944	14565	14517	21851
Mean intensity =	0.0	13.2	**0.3**	13.2	13.2	8.9	13.1	12.9	13.1
Mean int/sigma =	0.0	21.3	**1.3**	21.3	21.5	14.6	21.2	21.3	21.4
**Lattice type:B chosen** Volume:10357.03									

**Table 4:** A search for higher metric symmetry in SAXDUR using *XPREP*. Tetra­gonal-*I* was dismissed, in part due to its much higher *R*_sym_. All four remaining cells are pseudo-tetra­gonal. *C*-centred equivalents of the monoclinic-*I* cells are not shown

Option A:	FOM = 0.077 deg. TETRAGONAL I-lattice R(sym) = 0.195 [5588]
Cell:	14.688 14.727 15.244 90.04 90.03 89.99 Volume: 3297.48
Matrix:	-1.0000 -1.0000 0.0000 -1.0000 0.0000 -1.0000 0.0000 -1.0000 -1.0000
	
Option B:	FOM = 0.050 deg. ORTHORHOMBIC I-lattice R(sym) = 0.119 [5445]
Cell:	14.688 14.727 15.244 89.96 90.03 90.01 Volume: 3297.48
Matrix:	1.0000 1.0000 0.0000 1.0000 0.0000 1.0000 0.0000 1.0000 1.0000
	
Option D:	FOM = 0.030 deg. MONOCLINIC I-lattice R(sym) = 0.104 [3620]
Cell:	14.727 14.688 15.244 90.03 90.04 89.99 Volume: 3297.48
Matrix:	1.0000 0.0000 1.0000 1.0000 1.0000 0.0000 0.0000 1.0000 1.0000
	
Option F:	FOM = 0.044 deg. MONOCLINIC I-lattice R(sym) = 0.099 [3615]
Cell:	14.688 14.727 15.244 89.96 90.03 90.01 Volume: 3297.48
Matrix:	1.0000 1.0000 0.0000 -1.0000 0.0000 -1.0000 0.0000 1.0000 1.0000
	
Option H:	FOM = 0.050 deg. MONOCLINIC I-lattice R(sym) = 0.106 [3625]
Cell:	14.688 15.244 14.727 90.04 90.01 89.97 Volume: 3297.48
Matrix:	1.0000 1.0000 0.0000 0.0000 -1.0000 -1.0000 -1.0000 0.0000 -1.0000

**Table:** 

(*a*) Systematic absences for orthorhombic-*I* (option B):									
Systematic absence exceptions:									
	b--	c--	-c-	-a-	--a	--b			
N	417	417	368	368	459	459			
N I>3s	2	2	2	2	6	6			
<I>	0.3	0.3	0.3	0.3	0.4	0.4			
<I/s>	0.5	0.5	0.5	0.5	0.5	0.5			
Identical indices and Friedel opposites combined before calculating R(sym)									
Option	Space Group	No.	Type	Axes	CSD	R(sym)	N(eq)	Syst. Abs.	CFOM
[A]	**Ibca**	#73	centro	1	14	0.119	5445	0.5 / 4.4	9.67

**Table:** 

(*b*) Systematic absences for monoclinic-*I* (option F, lowest *R*_sym_):									
Systematic absence exceptions:									
	**-a-**								
N	368								
N I>3s	2								
<I>	**0.3**								
<I/s>	**0.5**								
Identical indices and Friedel opposites combined before calculating R(sym)									
Option	Space Group	No.	Type	Axes	CSD	R(sym)	N(eq)	Syst. ABS.	CFOM
[A]	**I2/a**	15	centro	1	3696	0.099	3615	0.5 / 4.4	3.00
[B]	**Ia**	9	non-cen	1	566	0.099	3615	0.5 / 4.4	5.40

## Appendix A Manual construction of a SHELXL ‘HKLF 5’ format datafile for SAXDUR

The *SHELX* ‘HKLF 5’ format for twinned diffraction data is well described in the *SHELXL* manual and elsewhere (see, *e.g.* the supporting information for Sevvana *et al.*, 2019 ▸). Briefly, each individual is assigned a separate batch number and twin-related reflections are grouped together sequentially. Batch numbers are set negative except for the last member of each group, for which the batch number is positive. There is no best way to generate such an ‘HKLF 5’ format datafile, but equivalents should be already merged because the format mandates suppression of merging within *SHELXL*. One could write a short program or script (*e.g.* Bolte, 2004 ▸), but the task itself is straightforward and does not require programming skills beyond text file reformatting. The following describes a logical step-by-step approach to generate an ‘HKLF 5’ format datafile from an ‘HKLF 4’ format datafile. It uses only common *unix* command-line tools *awk* and *paste* [these are available on *Windows via* the *Cygwin* project (Cygwin, 2020 ▸)] and is adaptable to other cases. For the pseudo-tetra­gonal twinning of SAXDUR in Section 5 above, the four individuals of the twin are related by successive rotation of 90° about the *c*-axis of a pseudo-tetra­gonal *I*-centred supercell. A positive rotation of 90° (anti­clockwise about *c*) is achieved by the following matrix:

Repeated application generates four matrices. Starting with the identity matrix, these are:

**A1. Split data into separate components**

For each of the four individuals, write separate files with indices transformed by matrices *A*2–5 using *awk*. The order does not matter, but a logical approach is to go stepwise about the (pseudo) fourfold. From a command-line terminal, issue the following commands:

awk ’{print $1,$2,$3,$4,$5,1,NR}’ monI.hkl > step1-1.txt

awk ’{print -$2,$1,$3,$4,$5,2,NR}’ monI.hkl > step1-2.txt

awk ’{print -$1,-$2,$3,$4,$5,3,NR}’ monI.hkl > step1-3.txt

awk ’{print $2,-$1,$3,$4,$5,4,NR}’ monI.hkl > step1-4.txt

In the above, the matrix transformation is accomplished by manipulation of the fields ($1, $2, $3) within the print commands. Also note that a batch number (1–4) and the line number (NR) of each reflection are appended to each line in the output text files. As stated above, in an ‘HKLF 5’ file, the last member of a group of twin-related reflections needs a positive batch number, with the rest negative. That will be fixed in a later step using these additional fields.

**A2. Combine components**

The individual files must be combined, with lines inter­laced in sequence. An easy way to achieve this is with the *unix* utility *paste*, which does a ‘parallel merge’. To trick *paste* into putting each component on consecutive lines, the delimiter is changed from the default (tab) to a newline (\n) character:

paste -d"\n" step1-1.txt step1-2.txt step1-3.txt step1-4.txt > step2.txt

The above writes a file in which contributions to each measured intensity are grouped, with sequential batch numbers.

**A3. Transform from monoclinic-*I* to monoclinic-*P***

Transformation from the *I*-centred setting to primitive monoclinic is *via* the following matrix:

This is the same matrix suggested by ADDSYM in Section 5.4 (Note: for other twinning cases, this matrix would be different). To apply it, again use *awk*:

awk ’{print ($1+$3)/2,-$2/2,($1-$3)/2,$4,$5,$6,$7}’ step2.txt > step3.txt

The index transformation inevitably writes some lines that have non-integer indices for each individual, which must be discarded.

**A4. Eliminate non-integer indices**

The following line of *awk* only accepts lines that have two dot ‘.’ characters.

awk -F. ’NF==3’ step3.txt > step4.txt

In the above, the *field separator* is first changed to a ‘.’ character. Lines in the input file are only transferred to the output file if the number of *fields* is 3. This works here because an ‘HKLF 5’ file should only have two decimal points [in the *F* ^2^ and *σ* (*F* ^2^) fields] per reflection. [Note, however, some *hkl* files written by *PLATON* store *F* ^2^ and *σ*(*F* ^2^) as integers, and thus should have no decimal points at all, and would require a modified condition (*i.e.* ’NF==1’) in the above *awk* command.]

**A5. Flag last member of each twin-related group**

The batch number of the last member of each group of twin-related reflections must be positive and the rest negative. The following *awk* one-liner gets part way there:

awk ’NR==1{printf "%s", $0; next}; {print " " $NF; printf "%s", $0}’ step4.txt > step5.txt

The above writes a new file with each line appended with an extra field containing the (original) line number of the next reflection in the file.

**A6. Generate ’HKLF 5’ format datafile**

The final step sets batch numbers for the last member of each group positive and the rest negative:

awk ’{if ($(NF-1)==$(NF)) {m=-1} else {m=1}; printf "%4d%4d%4d%8.2f%8.2f%4d\n", $1,$2,$3,$4,$5,$6*m}’ step5.txt > step6.hkl

In the above, (original) line numbers of consecutive reflections are compared and their batch-number signs modified, as per the ‘HKLF 5’ format rules. Lastly, it writes an *hkl* file formatted as ‘3I4, 2F8.2, I4’. The fastidious might want to manually edit the file termination line(s), but that is not necessary for proper *SHELXL* operation. For convenience, the above commands are available in the supporting information as a plain text file, suitable for copy/paste. They may also be combined into a single script along with additional commands to delete inter­mediate files.
